# Poster Session I - A144 PATIENT AND HEALTHCARE PROVIDER VOICES – LISTENING TO IMPROVE: EVALUATING A MULTIDISCIPLINARY COORDINATION OF CARE PATHWAY FOR PANCREATICOBILIARY CANCER

**DOI:** 10.1093/jcag/gwaf042.144

**Published:** 2026-02-13

**Authors:** P Mathura, E van der Raadt, A Kwan, K Blackie, M Timmermans, G Buchanan, A Nordin-Garrett, J Nilsson, S Wasilenko, S Perryman, S Veldhuyzen Van Zanten, D Bigam, J Shapiro, K Dajani, B Anderson, C Fung, G Sandha

**Affiliations:** Alberta Health Services, Edmonton, AB, Canada; University of Alberta Faculty of Nursing, Edmonton, AB, Canada; Alberta Health Services, Edmonton, AB, Canada; Alberta Health Services, Edmonton, AB, Canada; Alberta Health Services, Edmonton, AB, Canada; Alberta Health Services, Edmonton, AB, Canada; Alberta Health Services, Edmonton, AB, Canada; University of Alberta College of Health Sciences, Edmonton, AB, Canada; University of Alberta College of Health Sciences, Edmonton, AB, Canada; Alberta Health Services, Edmonton, AB, Canada; Medicine, University of Alberta, Edmonton, NS, Canada; University of Alberta College of Health Sciences, Edmonton, AB, Canada; University of Alberta College of Health Sciences, Edmonton, AB, Canada; University of Alberta College of Health Sciences, Edmonton, AB, Canada; University of Alberta College of Health Sciences, Edmonton, AB, Canada; University of Alberta College of Health Sciences, Edmonton, AB, Canada; University of Alberta College of Health Sciences, Edmonton, AB, Canada

## Abstract

**Background:**

The Edmonton Pancreaticobiliary Inflammation and Cancer (EPIC) Program is a nurse navigator (NN) led multidisciplinary coordination of care pathway aimed at improving care coordination, access to supportive services, and clinical outcomes for patients with pancreaticobiliary cancer.

**Aims:**

Examine the perspective and experience of patients, their families, and healthcare providers (HCPs) in the EPIC program.

**Methods:**

A mixed-methods design was used. Patient feedback was collected through a paper survey (N = 90), structured interview (N = 119), and NN notes documenting patient comments (N = 15). All patients and family members were eligible to participate. An online survey was distributed to a purposive sample of HCPs (N = 24) involved with the EPIC program. Quantitative survey data were analyzed descriptively, while qualitative data from interviews, nursing notes, and open-ended survey responses underwent thematic analysis.

**Results:**

Patient feedback from surveys (n = 41, 46%), interviews (n = 63, 53%), and nursing notes (n = 15) generated nine themes. Overall, 85% of patients reported high satisfaction, citing emotionally supportive care, timely communication, effective coordination and navigation, improved access to investigations and symptom relief, and clear information provision. Among HCPs (n = 12, 50%), 83% felt the program met patient needs, 100% agreed multidisciplinary coordination improved, and 92% felt comfortable accessing the program. Positive program experiences included dedicated contact personnel, timely communication, and access to NN and dietitian. Patients valued emotional, medical, and resource-based support, while HCPs emphasized timely coordinated care, streamlined communication, and rapid access to referrals and investigations. Patient-suggested improvements included more written education materials, increased in-person contact, and extended program hours. HCPs recommended raising program awareness, simplifying referrals, funding to expand NN staffing, and broadening the scope to include additional hepatobiliary cancers.

**Conclusions:**

The EPIC Program is highly valued by patients and HCPs. The findings strongly support continued investment in NNs, communication infrastructure, and program expansion to strengthen pancreaticobiliary cancer care.

**Funding Agencies:**

None

